# Dancing with death. A historical perspective on coping with Covid‐19

**DOI:** 10.1002/rhc3.12225

**Published:** 2021-05-18

**Authors:** Beatrice de Graaf, Lotte Jensen, Rina Knoeff, Catrien Santing

**Affiliations:** ^1^ Faculty of Arts Utrecht University The Netherlands; ^2^ Faculty of Arts Radboud University Nijmegen The Netherlands; ^3^ Faculty of Arts University of Groningen Groningen The Netherlands

**Keywords:** history of crisis management, pandemics, social adaptation

## Abstract

In this paper, we address the question on how societies coped with pandemic crises, how they tried to control or adapt to the disease, or even managed to overcome the death trap in history. On the basis of historical research, we describe how societies in the western world accommodated to or exited hardship and restrictive measures over the course of the last four centuries. In particular, we are interested in how historically embedded citizens' resources were directed towards living with and to a certain extent accepting the virus. Such an approach of “applied history” to the management of crises and public hazards, we believe, helps address today's pressing question of what adaptive strategies can be adopted to return to a normalized life, including living with socially acceptable medical, hygienic and other pandemic‐related measures.

## 摘要

本文研究了一个问题，即历史上社会如何应对大流行危机、如何试图控制或适应疾病、或甚至努力战胜死亡陷阱。基于历史研究，我们描述了过去四百年里西方社会如何适应或摆脱困境和限制性措施。特别地，我们希望了解历史上公民资源如何被用于适应病毒，并在一定程度上接受病毒。我们认为，对危机和公共危害管理采取这样的“应用史”（applied history）方法，能帮助应对当前的迫切问题，即能通过什么样的适应性策略回归正常生活，包括适应社会认可的医疗、卫生及其他与大流行相关的措施。

## Resumen

En este artículo abordamos la cuestión de cómo las sociedades enfrentaron las crisis pandémicas, cómo intentaron controlar o adaptarse a la enfermedad, o incluso cómo lograron superar la trampa mortal de la historia. Basándonos en la investigación histórica, describimos cómo las sociedades del mundo occidental se adaptaron o salieron de las dificultades y las medidas restrictivas durante los últimos cuatro siglos. En particular, estamos interesados en cómo los recursos de los ciudadanos históricamente arraigados se dirigieron a vivir con el virus y, hasta cierto punto, a aceptarlo. Creemos que este enfoque de “historia aplicada” a la gestión de crisis y peligros públicos ayuda a abordar la urgente cuestión actual de qué estrategias de adaptación se pueden adoptar para volver a una vida normalizada, que abarque vivir con servicios médicos y de higiene socialmente aceptables y otras medidas relacionadas con la pandemia.

## SETTING THE SCENE: THE ‘DANSE MACABRE’

In 1851, German artist Alfred Rethel (1816–1859) made an allegorical drawing of cholera raging through Paris. Rethel's drawing is based on Heinrich Heine's dramatic account of a disastrous cholera outbreak in Paris (1832) for the Augsburg *Allgemeine Zeitung*. The image represents Heine's description of the appearance of cholera at a masked ball: Three dancers suddenly fall dead on the floor, at the entrance of Death, who is playing an imaginary violin made of crossbones. The other dancers flee the room, whereas Cholera, dressed as an Egyptian mummy sits on the throne carrying the scourge of disease as a scepter (Hertel, 2000).

**Figure 1:**
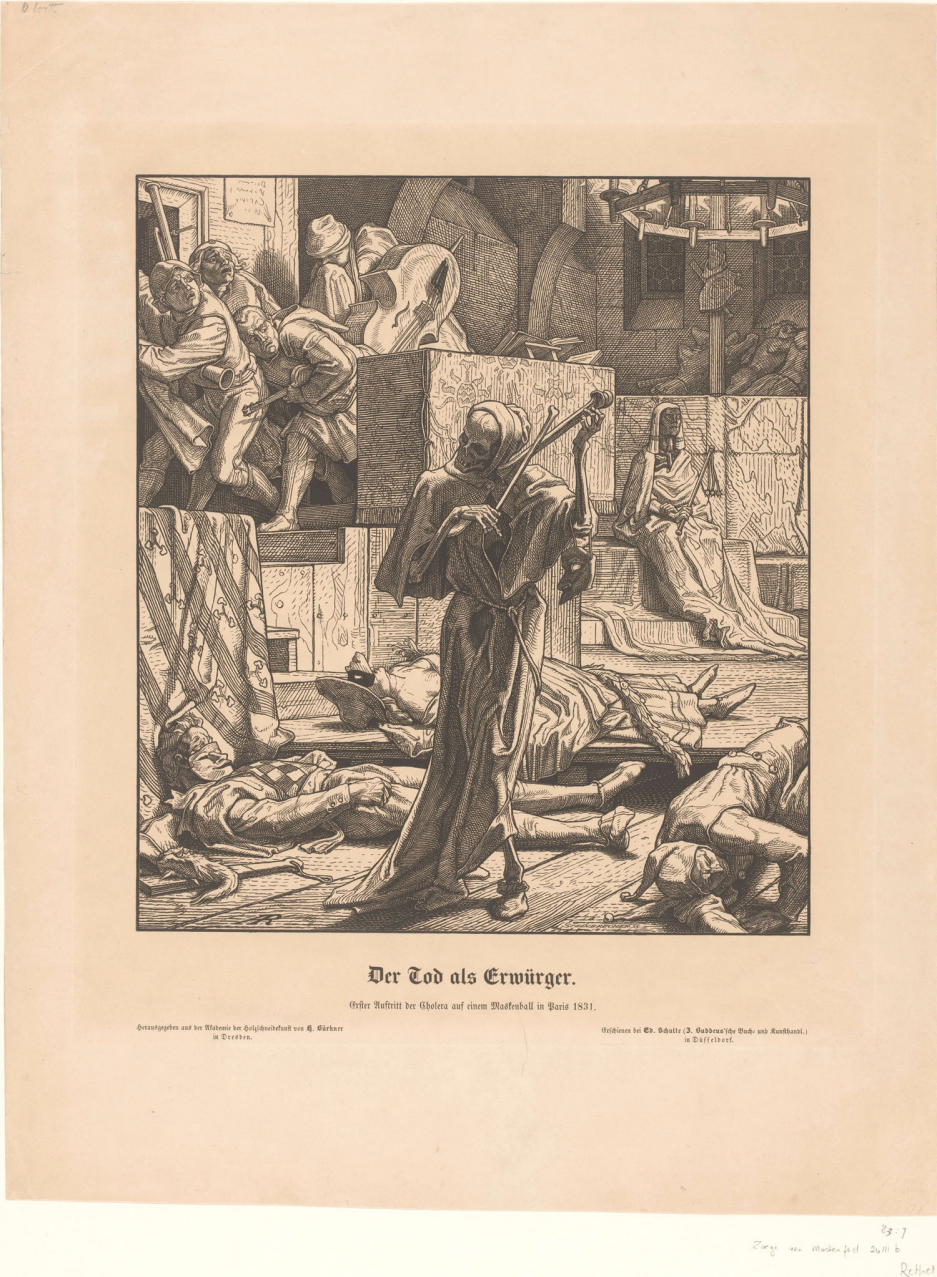
Alfred Rethel, “Der Tod als Erwürger, Erster Auftritt der Cholera auf einem Maskenball in Paris.” Published by Ed. Schulte in Dresden in 1851. Rijksmuseum, Amsterdam

**Figure 2:**
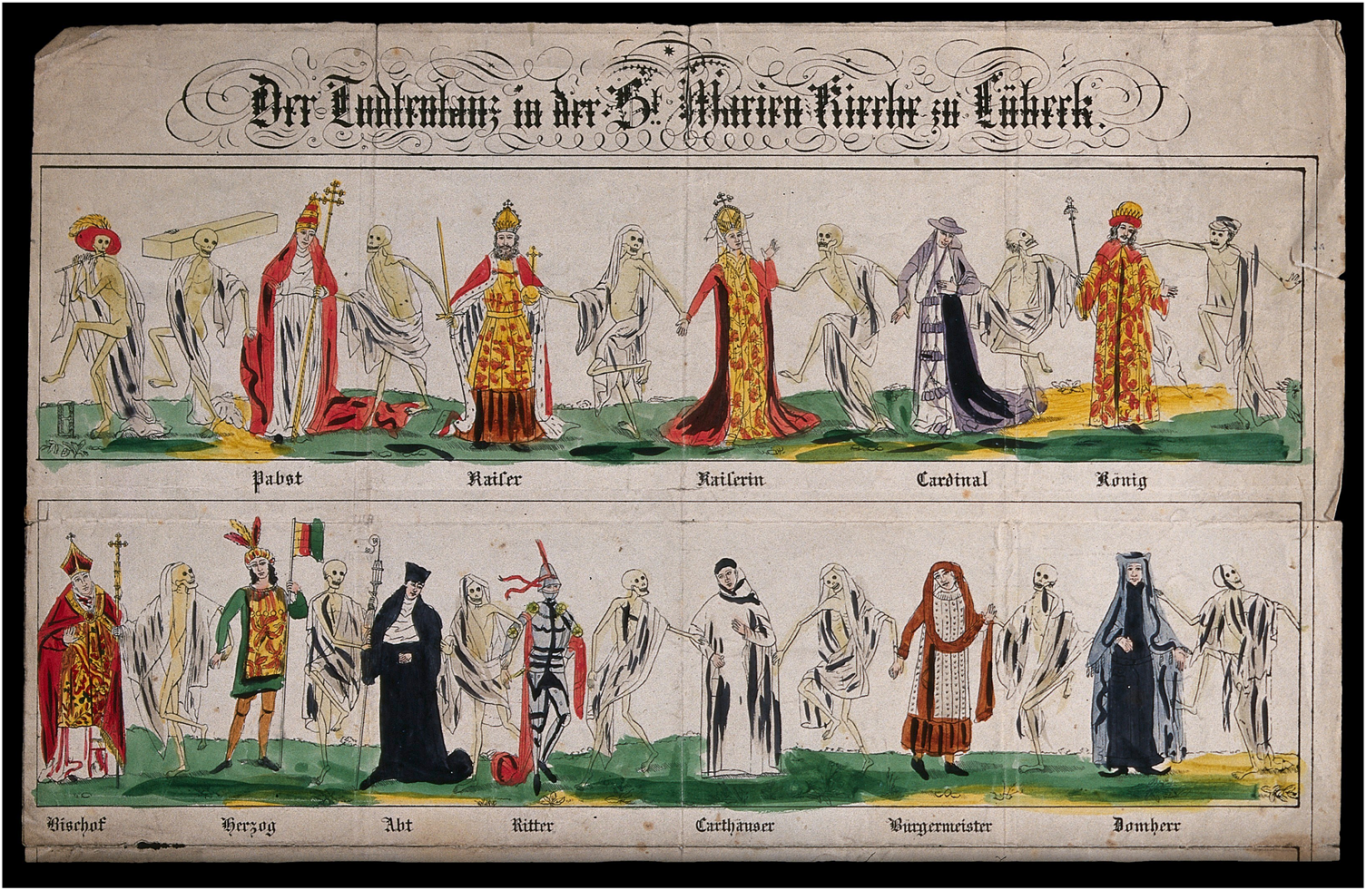
Dance of Death painted on the walls of the “chapel of the dead” on the north side of St. Mary's church at Lübeck. All four walls of the chapel were covered with paintings of the Dance, so whoever entered the chapel found himself in the center of the dance and almost literally became part of it. Courtesy: Wellcome Images

**Figure 3:**
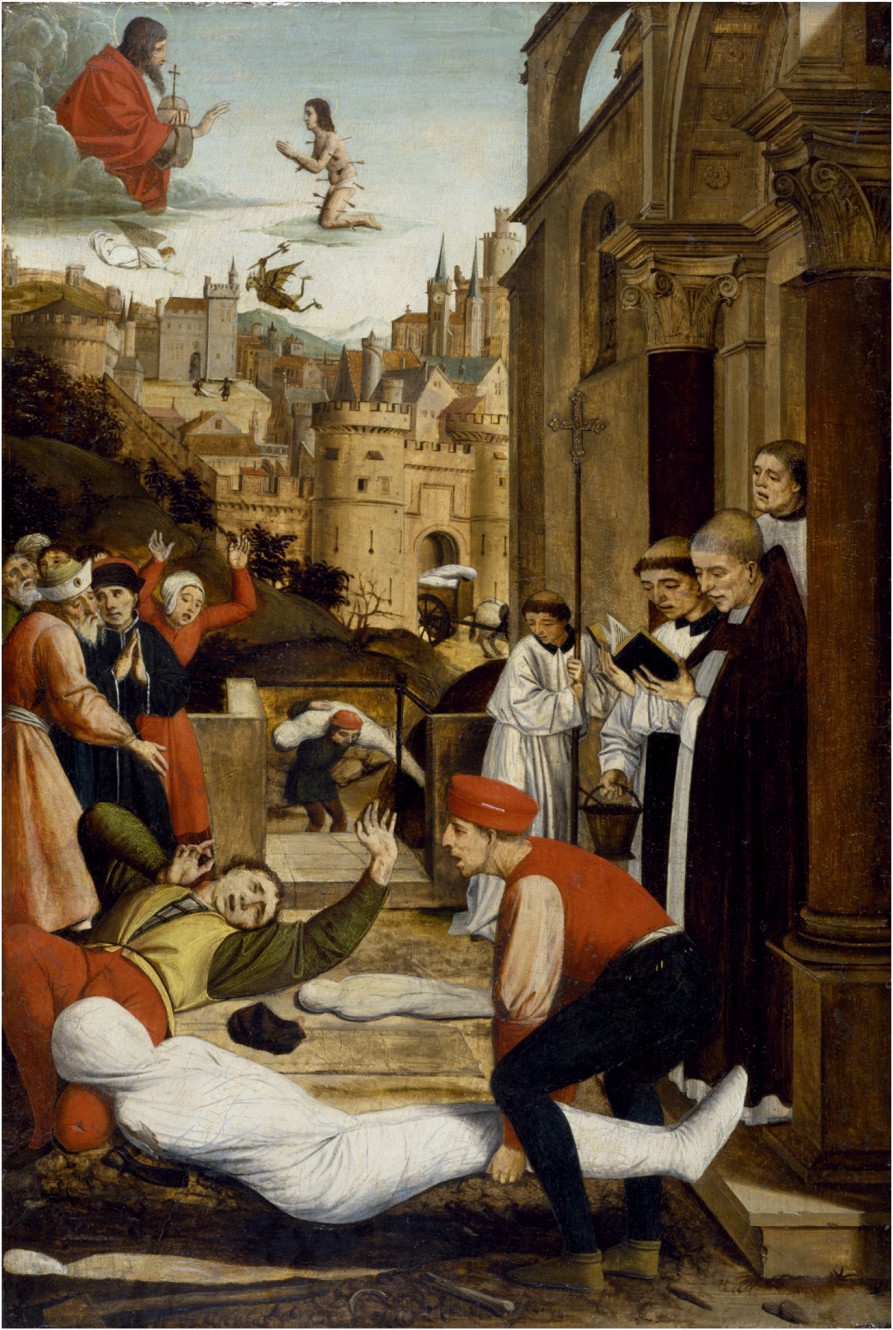
Josse Lieferinxe Altarpiece

**Figure 4:**
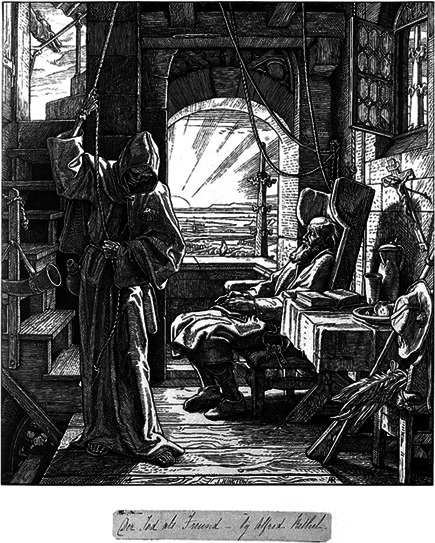
Alfred Rethel, *Death as a Friend*, 1851. Courtesy: Wellcome Images

Rethel's drawing belongs to a centuries‐old cultural tradition of “the danse macabre.” Born from the smoking debris of the medieval plague, it represents the idea that although societies are plagued by inequity, still everyone, rich or poor, man or woman, wise or ignorant, is equal in death: “Death comes equally to us all, and makes us all equal when it comes,” to quote the English poet and scholar John Donne (Binski, 1996; Dreier, 2010). Dances of death tableaux reminded the viewer of the transience of life, the relentlessness of death, and the moral obligation of charity and solidarity. Thus, the danse macabre was as much a social critique as a direct representation of vulnerabilities and entangled societal issues like disease, war, poverty, and famine. During the Middle Ages dances of death were found on cemetery walls, in churches, and on street corners (Figures 1–4).

Remarkably, dances of death have remained familiar motifs in imagery and poetry up until today and have even resurfaced in medical and political debates on how to tackle Covid‐19. The engineer and journalist Tomas Pueyo, for example, argues that governments, after applying the hammer, must learn how to “dance with the virus.” As a result, the dancing metaphor has become synonymous with the question of how to and when to strengthen or loosen lockdown measures (Pueyo, 2020a, 2020b), including the introduction of “basic dance steps” that countries should follow to effectively exit the crisis mode into a (more) normal situation (Pueyo, 2020c). Pueyo's technocratic and top‐down choreography comprises social‐distance measures, intensive contact tracing, extensive public education on hygiene, banning large gatherings, and an interplay between the removing and tightening of restrictions. Yet, unlike the historical metaphor, it pays little heed to the social inequities of the covid‐19 crisis. As such it fits a *biomedical* understanding of epidemics, focused on campaigns against specific microbiological agents, but with very little attention for the socioecological determinants of disease and the historically rooted coping mechanisms of any society. (Snowden, 2020; Ziegler, 2015).

In this paper, we offer an array of examples from history on how people dealt with past pandemics. In doing so, we realize that today's day and age with its technological and medical inventions is a different country compared to earlier periods in history. Yet, we do believe that in times of pandemic crises—such as today—it can be helpful to put context to those medical interventions, and show how people dealt with epidemics before antimicrobial drugs and vaccinations were discovered. On the basis of these past histories, we present an alternative choreography, a different series of dancing steps, which are rooted in history and based on social and cultural *coping mechanisms*.

In particular, we are interested in how historically embedded citizens' resources were directed towards living with and to a certain extent accepting the virus. Such an approach, we believe, helps to contextualize today's health and vaccination strategies and addresses the pressing question of what adaptive strategies can be adopted to return to a normalized life, including living with socially acceptable medical, hygienic, and other pandemic‐related measures. With our historical approach, we first and foremost draw attention to initiatives in the past that have arisen, bottom‐up, out of society itself. Thus, rather than focusing on *governments* and their top‐down interventions alone, we address the question of how *societies* coped with pandemic crises, how they tried to control and adapt to the disease, or even managed to overcome the death trap. In line with Marston et al. (2020), we address historical examples on how “meaningful relationships between communities and providers were nurtured to ensure sustainable and inclusive participation,” and we also show what happened when these meaningful relations did not emerge and community participation did not work.^1^ In other words, we address the question of how people either accommodated to or exited hardship and restrictive measures. This question, and the accompanying argument, is in line with the British Academy's recent response to Covid‐19 that pointed to

… the vital importance of *community‐led responses* that draw upon local knowledge and resources, and build capacities and channels of interconnectedness between government, community organizations and the public. The evidence clearly shows that those communities that entered the pandemic with such infrastructure in place have been in the best position to respond (The British Academy, 2021).

Thus, our paper responds to these and other recent calls to look back at past epidemics to develop community engagement strategies within the COVID‐19 response (Gilmore et al., 2020), and to come to a new understanding of dealing with pandemics (Brands & Gavin, 2020). For this reason, our approach to Covid‐19 is a historical (and qualitative) approach. This also means that our paper has a narrative structure. It traces the historical trajectory, identifies three modes of dealing with pandemics on a sociopsychological and sociological plane (see below), and translates these into modes of orientation for present‐day approaches.

Our paper draws from empirical material that we collected in our research, spanning the medieval up until contemporary history. Most examples stem from a Western, or Dutch, setting. Yet, they highlight societal dynamics that are applicable more broadly. We do acknowledge that the way epidemics play out depends on local, social, and cultural contexts. Yet, societal responses to epidemics reveal striking similarities across countries and historical periods. That is why, we argue, local examples can be used to identify broader concerns and strategies, especially if we connect them to secondary literature on other cases (cf. Brands & Gavin, 2020, pp. 1–21; Green, 2015, p. XV; Jackson, 2018).

## THE MERIT OF A HISTORICIZING BROAD APPROACH TO PANDEMIC CRISES

Before discussing coping and adaptive strategies in history, it is necessary to first elaborate on our historicizing approach and research design. Why do we feel that history could provide us with insights, when the way the pandemic is battled today—with highly centralized governments, cutting‐edge medical knowledge of virus and vaccines—is so novel, and dates back only to the last couple of decades? How does it serve our present‐day purposes to know more about how citizens in the early 20th and 19th centuries or in even medieval times dealt with pandemic situations? There are at least two reasons for taking historical lessons seriously.

First of all, a pandemic was never only about viruses and bacteria—something governments and public opinion often seem to forget. In the long course of history, our current understanding of a pandemic as a microbiological phenomenon is very new. It is bound up with the laboratory identity of disease and with the idea that a disease is caused by a specific disease agent (a virus or bacteria) (Cunningham, 1992; Last, 2001; Snowden, 2020). Only since the late 19th century was the term “pandemic” coined, in a House of Lords speech of 1883 (Playfair, 1883, pp. 1020–21). Since then, the discovery of microbiological agents brought about a strong emphasis on policy frameworks that predominantly focus on infectious disease management. Yet, medical interventions alone always fall short in dealing with pandemics. Even the World Health Organisation is well aware of that. In April 2020 (barely 6 months after the virus was first identified), the WHO has formulated conditions for lifting coronavirus lockdown measures that markedly involve not only medical criteria (such as the contagion rate, number of positive tests, etc.), but also touch upon social, economic, psychological and educational aspects (World Health Organization, 2020). Yet, even although the WHO has acknowledged the need for a broad approach, it has not been translated enough in encompassing structures (Brands & Gavin, 2020). The WHO still follows a strict, top‐down trajectory, with a remarkable lack of attention for culturally embedded coping strategies that may already be in place. That is the reason why we feel a historicizing approach and a highlighting of forgotten, but effective, social adaptive strategies from the past has great merit for the current debate and needs to be reiterated over and over again.

The second reason for historicizing the struggle with pandemics in society is the fact that this lack of attention for historical, cultural, and social coping strategies constitutes a breach with the past in itself. From the Middle Ages onwards until far into the 19th century, citizens, societies, and governments always tried to learn from the past. For example, for centuries, epidemic measures were recorded in guidelines, memory books, almanacs, and town chronicles. They served as “aide‐mémoire” as well as “archived repertoires” and were annexed to the town hall archives of laws, statutes, and ordinances. The authorities used them as “dossiers” or “manuals” to orientate themselves and develop policies. When an epidemic threatened a town or region, these “archives of knowledge” were carefully consulted and used as (historical) guidelines (Bakker, 2020; Pollmann, 2016). The way epidemic policies were devised during the so‐called Second Plague Pandemic (between the mid‐15th and late 18th centuries) offers an excellent example of how this was done. At the time, plague was a discontinuous phenomenon with several silent phases. During every outbreak, authorities not only carefully recorded successful strategies but also identified and protocolled when and why measures failed. These records functioned as a collective memory, helping governments to devise adequate measures in future epidemics (Carmichael, 1998). History, in other words, was used as a compass for orientation in the future.

Hence, if we adopt a broader historical perspective on “exit strategies” and survey the past, we can discern three types of—what we delineate as “adaptive strategies”: Strategies adopted in society to attempt to *overcome or control the disease*, to *accommodate to* the epidemic or pandemic situation, or to *reform the system* as such.

(1) Social support for and a common articulated belief in the so‐called *magic bullet*: The one intervention expected to *neutralize* the entity causing the disease, thereby providing a very specific and targeted type of adaptive strategy.
(2) The adoption of social coping mechanisms, which are rooted in cultural practices, intended *to accommodate the pandemic*, to manage and mitigate the crisis and its fall‐out, by keeping society together by nonmedical means. These may include temporally and locally specific mobilization of citizens' resources around specific aspects of the crisis, often hinging on the search for new meaning and resulting in new instruments and institutions of solidarity.
(3) Attempts to overcome or control the crisis, through addressing the socioeconomic circumstances that exacerbate the effects of the crisis for certain groups by *campaigning for reform, organizing protest and demanding change of the system as such*. This third category often involved civil disobedience, protest, and rebellion, where “adaptive strategies” in themselves could lead to political crises, chaos and dissolution in turn, and the overruling of previous measures.

We will identify and reflect upon the way these adaptive strategies were deployed in history and will estimate what characterized successful adaptive strategies. With “successful adaptive strategies,” we follow definitions laid down by Comfort et al. (2010) and Boin et al. (2017) who have highlighted the social and cultural dimensions of adaptation. In line with their work, we ask which *adaptive strategies as strategies societies developed in history to cope with crisis, bounce back, and build upon crisis experiences—and what underlying mechanism can be discerned that explains their success*. As stated before, this is a narrative, qualitative historical investigative effort, not intended to produce statistical data or predictive models. Yet, with the historical context presented here, we hope to raise awareness for broader, creative approaches to pandemics. The strength of this “thinking with history” approach lies in the fact that it thus may open up scenarios and strategies that go beyond today's resources. As historian Monica Green has argued in her account of plague and Ebola: “With climate change, among other factors, pushing the homeostasis of our own world out of balance, the past—which is the only knowable guide we have to the future—merits revisiting” (Green, 2015, XVII). This echoes the words of public historian John Tosh that “history seldom affords a basis for prediction, but it feeds the imagination about the potential for alternatives in the future” (Tosh, 2008, p. 7).

In the following paragraphs, we will focus on the above‐delineated three types of adaptive strategies. We hope to present such “food for the imagination” and formulate a couple of general insights in the conclusion.

## MAGIC BULLETS

For a society to cope with epidemic or pandemic illnesses, a willingness to acknowledge the illness and accept the cure for it is paramount. Before the invention of vaccines, there was no straightforward medical way to eradicate epidemic or pandemic illnesses. Yet, in some places, a very specific type of remedy existed, namely, the social belief in divine intervention mediated by holy men or women or at least divine intervention by proxy: By kings or queens administering the “royal touch.”

The remedy of this “royal touch” was applied for example (but not uniquely) in cases of scrofula, a range of diseases loosely related to glandular disorders in the neck and face. In popular terminology this disease was known as *le mal le roy* or the king's evil—“evil” as in the Lord's Prayer, where *malum* simply means illness or malady (Barlow, 1980; Bloch, 1924, p. 4; Kipple, 1993, pp. 998–999). According to popular belief, it was curable by royal touch and indeed we have many accounts of people healed after being touched by the monarch (although often this was also attributed to the tombs of saints in the vicinity of the ceremony). To the taste of many Protestants, the procedure was too miraculous, but it proved immensely popular during the 17th century: The Stuart Kings attracted huge crowds seeking relief from the disease. The custom persisted and even experienced a revival in the late 18th century, with the French king Louis XVI touching 2400 people suffering from scrofula (McManners, 1999, p. 13–14; World History Commons, 2020). Or with the French emperor, Napoleon, himself, as depicted in the painting by Antoine‐Jean Gros in 1804 as a king of yore, administering the royal “touch” to a dying soldier during the Jaffa campaign of 1798–1799 (Grigsby, 2002, p. 65–103).

Interestingly, not the touch itself, but the subservience to it, the ascribed legitimacy and belief in the king as a divinely ordained ruler, explains the popularity of the ritual. And it is, therefore, not surprising that the practice was abandoned not so much with the rise of inoculation against various diseases since the late 18th century, but with the political and theological changes in the understanding of the monarchy after the French Revolution (with Napoleon's painting as an imperial hiccup), and even before that time, in the age of the Enlightenment. Still, traces of this popular belief in the “divinitus,” the divine gift to cure, could be argued to have resurfaced in popular belief since the 20th century in the ability of vaccines and doctors to “magically” make the disease go away. Here, as with the “royal touch,” the expectations, the symbolic value of the administration of the medications are high, but crucially hinge on the willingness of the populace to accept the legitimacy of the system: The monarch as divine ruler, or the medics as omniscient healers. And as with the “royal touch,” a vaccine in itself cannot make the pandemic go away—a willingness to come forward and accept the “magic bullet” is required, a disposition to suspend disbelief and communal support for the legitimacy of the cure. Given this historical backdrop, we argue that the belief in “vaccines” emerged as a modern‐day, highly focused adaptive strategy, yet that this “magic bullet” echoes the premodern custom of the “royal touch,” of a “holy sacrament” almost.

For at least a century, it has been generally agreed upon that the fastest and most effective way out of a pandemic is a vaccine, Covid‐19 being no exception to that rule. The greater that political conviction and desire, the sooner a coalition of scientists and pharmaceutical companies will be mobilized to indeed produce such a vaccine. This rock‐solid political faith in vaccines is rooted in the idea that since the very first vaccine against smallpox, developed by Edward Jenner (1749–1823) at the end of the 18th century, vaccines have worked as magic bullets, either immunizing large populations or eradicating deadly epidemics. The term “magic bullet” was first used by Paul Ehrlich (1854–1915) to describe the effective working of arsphenamine—patented as Salvarsan 606—in the treatment of syphilis. Since then, magic bullets have been described as biomedical interventions (mostly drugs) that could kill specific microbes (e.g., antibiotics), or supplement deficiencies (e.g., insulin), without harming surrounding tissues. Although Covid‐19 vaccines are preventive interventions and do not kill the disease agent, they are presented as classic magic bullets—as perfectly targeted, quite harmless, relatively simple interventions that promise immediate relief.

We could, of course, argue that vaccines against infectious diseases like smallpox, measles, rubella, mumps, and poliomyelitis, have been very successful. High levels of vaccination have guaranteed immunity and have greatly increased children's chances to survive into adulthood. Yet, historians have also been skeptical of this conclusion. Thomas McKeown, for instance, famously argued that from the 1770s the decline in mortality from infectious diseases and the consequent rise of populations must be attributed to better economic conditions and related improvements in diet and standards of living, rather than medical improvements in vaccination, sanitation, and quarantine (McKeown, 1976). Already, during the early years of the plague, it was obvious that death rates mostly depended on socioeconomic circumstances and political strategies (Curtis & Roosen, 2017; Roosen & Curtis, 2019).

Indeed, McKeown is right in raising the important question as to what determines a society's pattern of morbidity and mortality precisely (see also Colgrove, 2020; Szreter, 2001). Vaccination was never a straightforward affair, neither in the medical or in the sociopolitical sense and acceptance of it. Historians of medicine have often emphasized the complicated entanglement of medical and political discourses. Central to their histories is the close relationship among immunization programmes, the consolidation of the nation‐state, the peoples' sense of community and citizenship, and the profit driven interests of pharmaceutical industries (Aisenberg, 1999; Baldwin, 1999; Blume, 2017; Brunton, 2012). Most recently Paul Greenough, Stuart Blume and Christine Holmberg have argued that vaccination campaigns are political projects that depend and impact on social cohesion and trust (Greenough et al., 2017, p. 1–2). In other words, vaccines are not merely medical products. They are also the product of governmental legitimacy within society, as for a vaccine to be effective, it needs enough citizens who are willing to take it, and to “swallow” this medical sacrament administered to them.

To better understand this medical–sociopolitical conundrum, we turn to parliamentary and public debates on the Dutch Contagious Diseases Act in 1872. The debates followed a deadly smallpox epidemic in 1871, which killed over 15,000 people. According to parliament, the epidemic had been more lethal than cholera had ever been. Much of the discussion centered on an amendment of Article 17 obliging schools to refuse admittance to teachers and children without written proof of having had either smallpox or a vaccination. Under this new legislation entering schools without this “passport” was punished with a fine between 5 and 25 guilders (ca. 52–260 euro in current value) or a prison sentence of up to 3 days. The amendment was an important addition to earlier legislation from 1725, which only obliged local governments to promote and subsidize vaccination. In the debate, two important issues were at stake: (1) The fundamental right to religious and educational freedom and the liberal right to self‐government; and (2) the distinction between *temporary* emergency measures directed at containing an epidemic, and *permanent* preventive interventions like vaccines.

In contrast to England, where vaccination was made compulsory in 1840, vaccination was never made obligatory in the Netherlands. In fact, the English situation was represented as highly undesirable. An obligation was considered at variance with the fundamental right to religious and educational freedom and the liberal right to self‐government. Hence, vaccination was made only indirectly compulsory. Through obliging schools to refuse to admit unvaccinated children, the Dutch government kept citizens' rights to self‐determination intact (at least theoretically). It was generally believed that this solution was in accordance with the Dutch national character. The Dutch, so it was believed, generally dig their heels in when they feel pushed into decisions. So, coaxing, instead of coercion, was preferred to get people to vaccination stations (Opwyrda, 1873, p. 17).

Remarkably, the debates also touched upon the suitability of vaccination as a kind of “adaptive strategy”—the ability of a social system to bounce back during (pandemic) crises and build upon crisis experiences. Much of the debate centered on the distinction between temporary emergency measures directed at containing the disease and permanent preventive interventions like vaccines. It was generally felt that the Contagious Diseases Act belonged to the first category as it enabled direct measures to tackle an epidemic; for example, it gave local city councils more rights to intervene, it arranged matters pertaining to quarantine and it arranged safe burial of the dead. Vaccination, however, was seen as the odd amendment out. Although opponents of the amendment acknowledged the efficacy of the vaccine (smallpox vaccination in effect led to the eradication of smallpox in 1872), they argued that vaccination must be considered a prevention strategy rather than a medicine against the disease. Compared to quarantine measures, a vaccine would not immediately lead to a drop in infections. Influential Christian conservative politicians, therefore, argued that vaccination should not be part of temporary emergency packages (Opwyrda, 1873, p. 241). Instead, they proposed to regulate vaccination in a general public health act, a plea that was also taken up by other parliamentarians.

Underlying these debates on vaccination measures, principles regarding bodily integrity, self‐determination, and freedom of religion and education were at stake. These were considered “slow issues”—issues, that needed time to simmer before decisions could be made. Moreover, it was felt that such decisions should not be issued from the top down, but should emerge within society itself. Then, it was widely understood, that without social support and a sense of legitimacy, such central ordinances would not be efficient in any case. Today, we often tend to point to deep‐seated religious objections as *the* most important threat to vaccination campaigns. Yet, from the late 19th century, in particular after influential reformers wrote about the God‐given efficacy of Edward Jenner's pox vaccine, not the religious objections as such, but the intrusive, top‐down governmental measures aimed at overturning citizens' objections were considered the main obstacle. Conservative and many liberal representatives alike felt that the amendment unjustly interfered with citizens' consciences. They argued that no centralized (and secular) government had a legitimate right to intervene behind doors or even inside bodies.

In the end, common interest and the serious threat of a new epidemic carried the day. The second chamber approved the amendment by a vote 49 to 13, all the while acknowledging that “it is with a heavy heart that we cannot respect conscientious objections [of which there are many]” (Opwyrda, 1873, p. 278). Even so, the amendment was a particularly Dutch solution: Vaccination was not made compulsory; in theory, people could (and did) conscientiously object. Moreover, vaccination was not so much considered an “exit strategy,” ending an epidemic. Rather, parliamentarians insisted on considering vaccination as one among many other preventative and adaptive strategies and encouraged the government and society alike to find other ways and means to control the disease.

To wrap this part up, we started this part of the paper with the “royal touch,” a treatment that could only be successful when the population believed in it and was willing to accept it. As such, the touch not only cured the disease but also exposed the level of trust in and legitimacy of the monarch (MacMillan 2020, pp. 49–55). Although the magic of a vaccine is rooted in different rationality, it similarly relies on the willingness of the people to take it. This reverberated in Dutch 19th‐century worries over obligation versus consciousness and prevention versus cure. The importance of trust and legitimacy in rulers and their medicines also resounds in today's conscientious objections of antivaxxers as well in fears among the populace about possible side effects. In sum, from these historical narratives, it follows that authorities need to be careful with imposing vaccination measures. Surely, vaccination is paramount, yet at the same time, it is a preventative strategy that can never be fully successful without a bottom‐up approach that takes into account the (often historically rooted) expectations, worries, and objections in society.

## NO END IN SIGHT, BUT TRYING TO COPE

A vaccine clearly marks a *medical* ending to an epidemic and can be considered a highly focused, targeted adaptive strategy. Yet, it does nothing in terms of solving the underlying socioeconomic and socioecological problems that exacerbate a health crisis and work as breeding grounds for (new) viruses. A vaccine, in other words, fits a *biomedical* understanding of pandemic but does not satisfy syndemic approaches to the disease that build on a combination of biological, ecological, and socioeconomic factors that lead to social inequity and the unjust exercise of power (Horton, 2020; Singer, 2009).^2^ For this reason, it is valid to look back in time to historical *coping mechanisms*, ways to “live with the disease,” that encompassed broader adaptive strategies, intended to help society accommodate to a pandemic and in the end, hopefully, return to normalcy. What can we learn from such past strategies?

Most of these strategies rested on the assumption that epidemics would not simply disappear but rather be transformed into endemic diseases, “chronic” and periodically recurring illnesses, that affect people in different ways depending on local and social circumstances. Premodern understandings of epidemics viewed these diseases (*morbi epidemici*) as originating from the “constitution of the fatherland,” whereby “the fatherland” comprised an assemblage of ground, climate, seasons, a people's character, and ways of living (e.g., van den Bosch, 1778). An emphasis on local circumstances was apparent in premodern medicine but has since been removed from modern‐day biomedical approaches. Yet, interestingly, it has resurfaced in recent comparative plague research which identifies socioecological landscapes susceptible to epidemics (Crespo & Lawrenz, 2015).

Moreover, time and again history has shown that coping strategies were indispensable in a return to normalcy. Hygienic and social distancing measures usually helped for a while, but in the long run were not tenable for various socioeconomic reasons (Knoeff, 2020; Boylston, 2014). In times when plague broke out, approximately every 9 years, and when cholera also returned in waves every 15 years until the late 19th century, an epidemic was something citizens had to learn to live with. For instance, a city chronicle from the 14th century reports on the beneficial solemnity of the church bells ringing for the dead all day, and writes about making the best of this situation (Meinsma, 1924, p. 290) And yet, fortunately enough, at some point epidemics also disappeared. Hardly ever was this due to medical inventions. Charles Rosenberg, in his essay “What is an epidemic?” argued how “epidemics ordinarily end with a whimper, not a bang” (thereby referring to the famous poem, *The Hollow Men*, 1925, by T. S. Eliot). Quarantine, hygiene, and social‐distancing measures cannot be called new nor are these measures particularly “medical” (Rosenberg, 1989).

A more “holistic” or contextualized approach to epidemic or pandemic illnesses has already been touched upon in our introduction, with the cultural representation and dissemination of the “danse macabre.” This double‐edged attempt tried to acquaint the populace—via images, songs, and processions—with the ubiquity of death on the one hand, and on the other appealed to their better nature. Invoking the “danse macabre” always also was done to encourage people to better themselves in the face of death, and show solidarity and compassion to others. Samuel Cohn compounded this strategy in his study of epidemics from Antiquity up until the Ebola crisis in Africa in 2014. He distinguished various “mechanisms of unity”: Adaptive strategies intended to unite societies and inspire individuals and communities to extraordinary manifestations of compassion and abnegation (Cohn, 2018, pp. 2, 68–92). So, contrary to the sometimes‐held belief that periods of plague and pandemics only pitted people against each other, causing polarization and patterns of “othering,” Cohn postulates that epidemics far more often brought about unity and compassion. Epidemics oftentimes led to the bonding of groups, to the creation of trust networks, that were formerly divided by seemingly unbridgeable barriers:

… plagues, yellow fever in America and the Great Influenza of 1918–20 globally eased class, ethnic, sectional, and racial tensions and extended care and charity through the donation of resources and relief provided by priests, nuns, doctors, nurses, and others who often journeyed from distant places and died as a consequence of their charity (Cohn, 2018, p. 535).

Often these practices were directed at creating a shared sense of responsibility. For instance, during the Middle Ages, religious rituals dovetailed with down‐to‐earth care of the sick and needy. We can see this in Josse Lieferinxe's 1497 altarpiece for the church of Notre‐Dame‐des Accoules in Marseille, commissioned by a local religious confraternity. In the heavens, Saint Sebastian, pierced with plague arrows, is praying for humanity, while at the same time the members of the Sebastian confraternity on earth are diligently burying the dead.^3^ The altarpiece contradicts the myth that medieval plague victims were abandoned and left dying in the streets. On the contrary, church, as well as secular authorities, stimulated the performance of such grimy acts of mercy, including the commissioning of images and variations on the “danse macabre” theme. A similar combination of religious ritual and care for one's neighbor is visible in the work of Reformed writers. Luther wrote that “by God's decree the enemy has sent us poison. Therefore, I shall ask God mercifully to protect us. Then I shall fumigate, help purify the air, administer medicine, and take it” (Cunningham & Grell, 2001, p. 285). This was no empty rhetoric; Luther proved himself an example of Christian piety in converting his own house (a former monastery) into a makeshift hospital for plague victims (Luther, 1999, p. 119–138).

The trope of the confraternities, bands of peace activists, and “flagellants” militantly roaming through Europe, all the while publicly practicing their rituals of self‐mortification, returned in many more cultural representations. Flagellants spread their message of penance and called for reconciliation and solidarity during times of plague, from the 13th to the 15th century. Their appeals inspired a flourishing body of art, calling believers to convert, repent, and donate to the poor (Bornstein, 1993; Chen, 2018).

Jumping ahead in time, away from the orders and the (bloodied, religious) rituals into the 19th century, pandemic crises continued to inspire cultural representations and appeals to solidarity and charity. For example, in the aftermath of disasters in the Netherlands—be they floods, firestorms, or diseases—uncountable sermons, poems, paintings, songs, prints, and broadsheets appealed to a common (often even nationalist) sense of shared suffering as well as to a religious duty to help one's neighbor. These texts invoked standard argumentative schemes and topical metaphors, which encouraged the readers to care for one another. The combination of showing charity and being “truly Dutch” was mentioned so often in the context of disasters, that they became intrinsically linked (Jensen, 2021). Charity was also propagated in visual representations of disasters, such as paintings and prints (Asperen, 2020). In this way, authors and painters not only encouraged the Dutch people to help the victims but also shaped a sense of belonging together.

With every new disaster cultural media (literature, paintings, press, and pamphlets) emphasized the charitable nature of the Dutch people, and with success. Take, for example, the genre of songs: They not only played a significant role in spreading the news but also in offering consolation by expressing grief and compassion for the victims (Jensen, 2019). Furthermore, they functioned as educational tools by showing the audience how to cope with the dreadful events. Often, disasters were interpreted as signs of God's vengeance and as warnings that the listeners needed to repent to prevent new disasters. However, in other songs, more attention was paid to the grief of those who were left behind. National solidarity reached a peak in the 19th century when songs and concerts were also used as a means to raise money for the victims. During the cholera outbreak of 1832, for instance, a benefit concert was organized in the municipal theatre in Amsterdam to raise money for the cholera‐ridden (*Nederlandsche staatscourant*, November 6, 1832). In 1854, another huge event was organized in Rotterdam, with the participation of an orchestra with 100 musicians, a choir of 125 people, and the well‐known Dutch poet Hendrik Tollens (*Nieuwe Rotterdamsche Courant*, January 28, 1854). A total sum of 800 guilders (ca. 7500 euro in current value) was raised (Tollens, 1857).

Initially, charity was organized incidentally and on a local scale, but this changed during the 18th century when civil committees started cooperating with the local authorities. Churches also continued collecting money for victims, in particular, on prayer days. During the 19th century, the state started playing a more central role. The French king Louis Napoleon set the example by letting his administration coordinate the fundraising: Collections for the gunpowder explosion in 1807 and the major floods of 1808 and 1809 were immensely successful. This trend was continued in the 19th century, with every new disaster the “national enthusiasm” to donate grew. Cultural media, such as pamphlets, sermons, and songs, played an important role in reinforcing the legitimacy of royal rule, of mutual solidarity, and of prosocial behavior. They made use of a recurrent set of tropes, which linked the capacity for coping with severe disasters with the emergence of a Dutch national identity (Jensen, 2021).

This resulted in many a successful campaigns. During the 1849 cholera outbreak, for example, many collections were held to support the victims. In the coastal village, Scheveningen, the churches raised almost 3000 guilders and civic committees more than 8000 guilders—quite an impressive amount of money (ca. 33,000 and 87,000 euro in current value; *Opregte Haarlemsche Courant*, August 30, 1849. Similar activities were organized throughout the country. Publishers and authors also joined forces to raise funds. Several preachers published collections of songs, prayers, and sermons with the aim to collect money for the victims. After the outbreak of the cholera epidemic of 1866 King William III awarded medals to those who had shown exceptionally philanthropic behavior, a cultural practice copied from flood disasters.

In short, during the Middle Ages, the “danse macabre” inspired highly performative rituals, sending out a memento mori and calling for pacification, reconciliation, and compassion. In later ages, the rituals were less colorful, but the appeal to citizens to help their neighbors was as vociferous. We have focused here on the case of the Netherlands, but similar examples of selfless solidarity can be found in other communities across Europe and the United States. As Samuel Cohn has stated: “Past plagues ignited compassion, bringing volunteers to make sacrifices to complete strangers across class, ethnic, and racial divides” (Cohn, 2018, p. 557). Rebecca Solnit has moreover argued in relation to major disasters in the United States and Mexico that “the positive emotions that arise in those unpromising circumstances demonstrate that social ties and meaningful work are deeply desired, readily improvised, and intensely rewarding” (Solnit, 2009, p. 7).

As coping mechanisms and as adaptive strategy, such rituals, processions, and collections—in the entire variety of cultural forms—gave direction to suffering people. They pointed to a higher meaning and simply helped to alleviate the direst needs. Obviously, local communities also tried to uphold some sort of order in society by deploying these charitable strategies. The well‐to‐do citizens that founded the cholera committees in the first half of the 19th century, for example in Groningen, did so out of humanitarian and Christian considerations, but also to prevent bands of orphaned children and impoverished paupers from roaming the streets: To “liberate them from the humiliation of total poverty; and keep them as useful part of the civil society” (*Groninger Courant*, September 22, 1826; cf. Baron, 2006, pp. 394–396, 406–415). Such expressions of solidarity, of helping each other to cope with loss and trauma, left their traces in the cultural representation and helped society to accommodate to and preserve its order in the face of death and disease.

History offers countless more examples of how local and national communities, through cultural and religious practices, tried to adapt to crises by mobilizing in the name of solidarity (Bavel et al., 2020; Solnit, 2009, pp. 92–94). We, first of all, pointed to the variegation of cultural representations of plague and disaster, that helped people find a shared narrative and language in coping with loss and grief. These representations carried meaning in themselves, and mirror today's search for designing collective ways of mourning and finding rituals to cope with lives lost. Yet, in the second place, and on a more socioeconomic (or socioecological) plane, community engagement, in particular, proved to be a fundamental component of dealing with past epidemics—something that may feed into designing useful strategies for COVID‐19 responses today (Gilmore et al., 2020; Marston et al., 2020). This brings us to the third and last adaptive strategy: Organizing substantial reforms.

## PROTESTING THE SOCIOECONOMIC EFFECTS, ORGANIZING REFORMS

In addition to adaptive strategies aimed at stimulating charity, and accommodating a society to death and disease, epidemics and pandemics also functioned as catalysts of societal reform. The monumental late medieval dances of death reveal social protest by showcasing that, even though society is divided into social classes, before God all mortals are equal. Reform movements emerged in response to the inequity and injustices that epidemics laid bare, oftentimes ending in rioting and revolt. Such adaptive strategies were not aimed at instilling a spirit of endurance, conciliation and solidarity in the face of hardship. Neither were they directed at overcoming or controlling the disease. They were provoked by the perception of deep‐seated inequalities (including health inequity and measures perceived as unjust), exacerbated by the crisis.

For instance, in the period between 1200 and 1425, while Europe suffered from the devastating effects of plague, it saw no fewer than 1112 revolts. These revolts were not necessarily directly related to outbreaks of the plague, nor against the specific measures to contain those outbreaks. They were a protestation against the disproportionate impact of the disease on lower classes and on their social‐economic situation. In 1381, after the first wave of plague and against the backdrop of serious labor shortages, English peasants protested the continuation of their low wages. They demanded higher wages, and a higher place in the social hierarchy. They also demanded the abolition of serfdom and the granting of more political rights. Yet, at the same time, elites stuck to their privileged rights and tried to deny rewards to workers. Remarkably, those revolting hailed not only from the peasant population and the poorest classes as lower‐ranking merchant elites and people of a “middling sort” also participated in the rebellions against post‐plague tax increases. According to Cohn, these rebellions continued well into the 17th century, creating the systemic crisis of the *Bauernkriege* in the Reformation Era (Cohn, 2006, p. 208; Curtis, 2000).

However, not all protests and calls for reform ended in systemic violence and revolution—sometimes protests succeeded in mobilizing concrete reforms towards more justice and solidarity in society. In Northern Italy, equally afflicted by the plague in the 14th century, the peace movement of the Bianchi emerged. The Bianchi effectively compelled the various factions to engage in reconciliation and end political strife (Bornstein, 1993), creating new networks of trust and developing legitimate measures to alleviate the crisis. Particularly in the Netherlands, epidemics also gave rise to effective, bottom‐up movements for social reform. The 18th‐century charity initiatives mentioned above, were followed in the 19th century by local, nongovernmental charitable committees whenever the need arose. We can, of course, only speculate about the success of these local committees. Yet, the fact that the 19th‐century cholera riots across Europe, largely passed by the Netherlands, seems indicative of successful local approaches geared towards the particular needs of people in particular in the lower social economic classes. Such local initiatives increasingly put pressure on the nascent national government to launch centralized reforms and care programmes, independent from church and private initiatives (Baron, 2006, pp. 394–396, 406–215; Baron, 2021; Houwaart, 1991).

Attempts to reform and overcome the existing situation and improve social relations were not only directed by societal groups towards the authorities or the elites. Sometimes social campaigns were established to target their own constituents and citizens. The Bianchi movement mentioned above belongs in that category. Centuries later, similar campaigns were mounted. In 1838, Dutch preacher and philanthropist Ottho Gerhard Heldring (1804–1876) initiated a national fight against gin, which he considered a plague worse than cholera. Even more, he considered the two of them a toxic combination. Cholera, Heldring stated, “is a friend and companion of the ginman, he finds him in palaces, as well as in lowly cabins and huts” (Heldring, 1838, p. 11).^4^ Heldring supported his argument by careful calculations of gin consumption, taxes, death rates, and so forth. In 1836, the Dutch, so he concluded, had spent a stunning amount of 15 million guilders on gin (i.e., 160 million euros today). During the preceding cholera years between 1830 and 1836 the consumption of gin had increased by no less than 25%. Heldring concluded that had the people not spent all their money on gin, but on funds for medicines, funerals, care of the diseased, the orphaned, and widowed, society would have turned out so much better.

Again, from these various instances of social action and reform it becomes clear that epidemics not only stimulated patience, endurance, accommodation, and communal solidarity, but also functioned as a combustion chamber for protest movements. The less privileged grew so frustrated over inequalities and injustices exacerbated during pandemic times, that they took a leap forward and tried to change the system as such (van Bavel et al., 2020, p. 6).^5^

Oppositional tendencies like these—either directed against (local) governments or against fellow citizens—have to be taken into account when trying to steer out of the current crisis. The Leopoldina, the German National Academy of Sciences, therefore, rightfully stated in 2020 that citizens will accept and obey only those measures that are considered “clear, unequivocal, and sensible” (Leopoldina, 2020, p. 8). If they are considered haphazard, illogical, or even worse, unfairly distributed, society will rally against such measures. This pattern is compounded by historical evidence. Indeed, the present Covid‐19 crisis has already witnessed ample display of protest activity (we are writing this in December 2020) against lockdown and quarantine measures that are perceived as unjust and unnecessary (Censolo & Morelli, 2020). Not surprisingly, places where epidemics highlight inequalities experience a higher chance of outbursts of social protests (e.g., the Black Lives Matter protests in US cities). Equally worrisome is the level of conspiracy thinking that has risen in reverse relation to the level of trust in government. Also here, authorities would do well to keep the long history of epidemic rioting in mind. Not only is there a direct relation between such riots and a government's perceived performance in terms of just and transparent measures. There is also the fact that citizens can and should be involved, that protests may lead to and facilitate (local) reforms, thus succeeding in mitigating cleavages in society before they get out of hand.

## DANCING WITH DEATH: CONCLUDING FROM HISTORY

If we can glean anything in terms of the adaptive strategies from history presented in this paper, it may well be this insight: Social reaction to crisis and crisis measures very much reflects the way in which a crisis is handled transparently, consistently and in maintaining a fair and just distribution of the pain and suffering caused both by the pandemic and the measures to regulate it. Only under these conditions will “adaptive strategies” be considered legitimate, will they function as means to mitigate the impact of a crisis or overcome it, and will they enable a society “to build back better.”

Indeed, what follows from studying the three types of adaptive strategies laid out above, is that the question of *legitimacy*, hinging on trust and justice, is pivotal in explaining their success (or failure). Legitimacy was both crucial in understanding who spoke with authority and credibility in times of crises, and what measures were considered fair and just. Legitimacy worked through networks of trust rather than through centralized health programs up until the late 19th century. From the first type of strategy, the “magic bullet,” through the second, “finding unity” in and through suffering, to the third, “campaigning for reform”—forging mutual trust and legitimacy in society is crucial. If we want to perform a long‐lasting dance with the virus, we need to move beyond numbers and statistics. At the beginning of the Covid‐19 crisis, social anthropologist and renowned writer on African health, drought, and war crises, Alex de Waal argued that “although the pathogen may be new, the logic of social response is not, and it is here that we can see historical continuities” (de Waal, 2020). Three insights can be summarized here with respect to engendering legitimacy and trust in dealing with pandemics.

First of all: One way of forging communities of trust and solidarity were the slew of representations and rituals that were drafted and designed to find meaning in “living with the crisis together,” with the “danse macabre” being the most powerful of these. Yet, much of these citizens' resources to mobilize adaptive strategies through cultural representations and mediations, seems to have been lost in oblivion. Therefore, with this paper, we hope to contribute to a better understanding of the sometimes very cathartic and successful drama of such historical coping mechanisms.

In the second place, another insight that we can draw from history was also made by Charles Rosenberg: The fact that epidemics seem to follow a particular dramaturgic form with respect to societal reactions, and that a history of epidemics is, therefore, fundamentally a history of how societies cope with them:

Epidemics start at a moment in time, proceed on a stage limited in space and duration, follow a plot line of increasing and revelatory tension, move to a crisis of individual and collective character, then drift toward closure (Rosenberg, 1989, p. 2).

Tensions, calls for reform, or even protest will inevitably belong to the drama—the question is only how authorities and societies deal with them. The particulars of the drama (the measures taken, the way people react, structures of blame and the moral lessons afterwards) are grounded in the institutional forms and cultural assumptions of a particular society. We argue that understanding these cultural repertoires is essential in dealing with epidemic and pandemic situations. It is also essential in managing the dramatic cycle of an epidemic or pandemic and understanding when the cycle reaches the stage of protest.^6^ Current governments and societal groups alike could and should, therefore, invest in inventing and developing cultural and social rituals and symbols to cope with loss and grief (Jedan, 2020), something the Dutch association of mayors has taken up recently as well (Nederlands Genootschap van Burgemeesters, 2020).

Here, our third and final insight needs to be underlined—the importance of combining medical with social, compassionate interventions. One of the most powerful historical cultural repertoires that we have discussed here is the image of the danse macabre. This imagery was not just a cultural representation of death and disease, but was also always intended as specific type of adaptive strategy for a society in distress. Instead of focusing on managerial or engineering interpretations of the Dance (Pueyo's version), this paper argues to bring back the historical choreography, including parts of the medieval meaning of the danse macabre. This historical choreography of the dance was very much aware of the social critique that emerges in times of crisis. Death and disease come to us all, rich and poor, men and women, religious and secular. Yet, as the current epidemic also shows, some individuals and groups will always be hit harder than others. For this reason, the choreography should not only follow the medical steps in solving the epidemic conundrum, but should include (local) sociopsychological, socioeconomic and cultural steps as well. A Dutch committee—chaired by the mayor of Amsterdam—appealed to the government to do exactly that, invest in vulnerable groups, families at risk and set up local programs to support these groups (VNG, 2020). Historical examples support this initiative.

Rosenberg has argued that “plague (and epidemics in general) reminds us that human beings will not so easily escape the immanence of evil and the anxiety of indeterminacy. Mortality is built into our bodies, into our modes of behavior, and into our place in the planet's ecology” (Rosenberg, 1989, p. 17; see also Charters & McKay, 2020). For this reason we end this paper with the counterpart of the image of the introduction. In addition to representing death as a ruthless destroyer of life, Alfred Rethel also portrayed death as a friend, bringing release to the weary soul, and as a companion reminding us of our common destiny (Warthin, 1931). In the end, the historical motif of the “danse macabre” holds us all accountable: It questions the way we deal with our own possible death and disease. But also asks us how fairly and justly we, personally, and as a society, deal with those of others.

## ENDNOTES

1 This also indicates that we do not make sharp distinctions between formal governmental structures and society, since up until the late 19th century (or even the early 20th, before the emergence of the centralized welfare state), such distinctions did not make much sense. We do, however, discern between activities initiated by nonformal groups and bodies vis‐à‐vis those imposed by formal institutions in power.

2 Scholars from the humanities and social sciences have pointed at the entanglement of medical and societal approaches right from the start of the crisis. The concept of syndemics has be War I and warns for it with regard to Covid‐19, esp 45–48.

3 Meinsma (1924, p. 296–297) has an extract of archival findings and chronicles on the Plague for mainly the Low Countries which confirms this observation on the painting: https://art.thewalters.org/detail/6193/saint-sebastian-interceding-for-the-plague-stricken/, accessed March 22, 2021.

4 On 19th century societal reform movements see Janse (2007).

5 Already on January 30, 2020, Robert Peckham pointed to such an entanglement with regard to Chinese intervention in Hong Kong: “I've studied the history of viral outbreaks. Coronavirus panic shows how little China learned from SARS,” *The Independent*, January 30, 2020, https://www.independent.co.uk/voices/coronavirus-china-hong-kong-masks-public-health-disease-deaths-a9308616.html

6 Mac Millan 2020 analyzes this phenomenon for World War I and warns for it with regard to Covid‐19, esp 45–48.
